# Affective bias predicts changes in depression during deep brain stimulation therapy

**DOI:** 10.3389/fnhum.2025.1539857

**Published:** 2025-03-25

**Authors:** Brian Cui, Madaline M. Mocchi, Brian A. Metzger, Prathik Kalva, John F. Magnotti, Jess G. Fiedorowicz, Allison Waters, Christopher K. Kovach, Yvonne Y. Reed, Raissa K. Mathura, Camille Steger, Bailey Pascuzzi, Kourtney Kanja, Ashan Veerakumar, Vineet Tiruvadi, Andrea Crowell, Lydia Denison, Christopher J. Rozell, Nader Pouratian, Wayne Goodman, Patricio Riva Posse, Helen S. Mayberg, Kelly Rowe Bijanki

**Affiliations:** ^1^Department of Neurosurgery, Baylor College of Medicine, Houston, TX, United States; ^2^Department of Neurosurgery, University of Pennsylvania, Philadelphia, PA, United States; ^3^Ottawa Hospital Research Institute, University of Ottawa, Ottawa, ON, Canada; ^4^Nash Family Center for Advanced Circuit Therapeutics, Icahn School of Medicine at Mount Sinai, New York, NY, United States; ^5^Department of Neurosurgery, Carver College of Medicine, University of Iowa, Iowa City, IA, United States; ^6^Department of Psychiatry and Behavioral Sciences, Emory University School of Medicine, Atlanta, GA, United States; ^7^School of Electrical and Computer Engineering, Georgia Institute of Technology, Atlanta, GA, United States; ^8^Department of Neurosurgery, University of Texas Southwestern, Dallas, TX, United States; ^9^Department of Psychiatry, Baylor College of Medicine, Houston, TX, United States

**Keywords:** affective bias, facial emotion, mood proxy, deep brain stimulation, subcallosal cingulate, ventral capsule striatum, treatment-resistant depression

## Abstract

**Introduction:**

Deep brain stimulation (DBS) is a promising treatment for refractory depression, utilizing surgically implanted electrodes to stimulate specific anatomical targets within the brain. However, limitations of patient-reported and clinician-administered mood assessments pose obstacles in evaluating DBS treatment efficacy. In this study, we investigated whether an affective bias task, which leverages the inherent negative interpretation bias seen in individuals with depression, could serve as a reliable measure of mood changes during DBS therapy in patients with treatment-resistant depression.

**Methods:**

Two cohorts of patients (*n* = 8, *n* = 2) undergoing DBS for treatment-resistant depression at different academic medical centers completed an affective bias task at multiple time points before and after DBS implantation. The affective bias task involved rating the emotional content of a series of static photographic stimuli of facial expressions throughout their DBS treatment. Patients' ratings were compared with those of non-depressed controls to calculate affective bias scores. Linear mixed-effects modeling was used to assess changes in bias scores over time and their relationship with depression severity measured by the Hamilton Depression Rating Scale (HDRS-17).

**Results:**

We observed significant improvements in total affective bias scores over the course of DBS treatment in both cohorts. Pre-DBS, patients exhibited a negative affective bias, which was nearly eliminated post-DBS, with total bias scores approaching those of non-depressed controls. Positive valence trials showed significant improvement post-DBS, while negative valence trials showed no notable change. A control analysis indicated that stimulation status did not significantly affect bias scores, and thus stimulation status was excluded from further modeling. Linear mixed-effects modeling revealed that more negative bias scores were associated with higher HDRS-17 scores, particularly for positive valence stimuli. Additionally, greater time elapsed since DBS implantation was associated with a decrease in HDRS-17 scores, indicating clinical improvement over time.

**Discussion:**

Our findings demonstrate that the affective bias task leverages the inherent negative interpretation bias seen in individuals with depression, providing a standardized measure of how these biases change over time. Unlike traditional mood assessments, which rely on subjective introspection, the affective bias task consistently measures changes in mood, offering potential as a tool to monitor mood changes and evaluate the candidacy of DBS treatment in refractory depression.

## 1 Introduction

Treatment-resistant depression (TRD) is a form of major depressive disorder that does not respond to traditional treatments such as pharmacological intervention or cognitive behavioral therapy (Sackeim, 2001; Mayberg et al., 2005; Nemeroff, 2007). Those with TRD suffer from debilitating symptoms such as prolonged periods of depressed mood, low self-esteem, suicidal thoughts, and changes in sleep and appetite (Sackeim, 2001; Ionescu et al., 2015; Touloumis, 2021). With 2.8 million cases of TRD in the U.S. alone resulting in a total annual burden of $43.8 billion, there is an urgent need for new therapies that can treat TRD effectively (Zhdanava et al., 2021). Deep brain stimulation (DBS) is a relatively new method of treating TRD in which surgically implanted electrodes are used to chronically stimulate specific anatomical white matter targets within the brain and is thought to normalize activity across the circuit implicated in depressive disorders (Mayberg et al., 2005; Surguladze et al., 2005; Lozano et al., 2008; Malone et al., 2009; Harmer et al., 2009a; Heller et al., 2009; Crowell et al., 2019; Godlewska and Harmer, 2021). Although not consistent, DBS applied to the subcallosal cingulate (SCC) or the ventral capsule/ventral striatum (VCVS) has shown promising results in reducing depression severity in those with TRD (Mayberg et al., 2005; Lozano et al., 2008; Malone et al., 2009; Crowell et al., 2019; Drobisz and Damborská, 2019).

Quantifying the effects of DBS on mood has been a challenge (Daneshzand et al., 2018; Roet et al., 2020; Funkiewiez et al., 2004; Burn and Tröster, 2004; Temel et al., 2006; Campbell et al., 2012), in part related to limitations inherent to self-report measures. A major confounding hallmark of the self-report assessments for this disorder is the common occurrence of alexithymia in which patients cannot accurately quantify their emotional state in MDD (Taylor, 1984; Hemming et al., 2019). Effects of depression symptomatology are also compounded by demand characteristics and cost invasiveness in clinical studies, which can often influence patients to consciously alter their scores in order to suit the goals of the study (Davidson et al., 2020). Measures such as the Hamilton Depression Rating Scale [HDRS-17] or Montgomery-Asberg Depression Rating Scale [MADRS] are the current gold standard used to guide DBS programming (Hamilton, 1960; Wideman et al., 2013; Drobisz and Damborská, 2019; Davidson et al., 2020). While these assessments are accurate in assessing mood, they, and the clinical DBS trials that employ them, are limited by their reliance on a patient's insight into their own emotional state, which is often compromised in mood disorders such as depression (Davidson et al., 2020; Talarowska et al., 2016). In addition, as the field moves further in the direction of therapeutic settings tailored to the individual patient, there will be a need for metrics that can be assessed more frequently than the typical measurement window for clinician-administered scales which track changes in depression on the order of weeks to months (Harmer et al., 2009a; Lozano et al., 2019). Thus, the identification of an implicit measure of depression could allow for faster and more accurate identification of successful treatment paradigms and reduce variation in patient outcomes (Davidson et al., 2020; Fenoy, 2020; Dorz et al., 2004).

One promising measure of mood state is the construct of affective bias, the phenomenon whereby external emotional stimuli are interpreted in a manner consistent with one's own emotional state (Surguladze et al., 2005; Heller et al., 2009; Roiser et al., 2012; Harmer, 2013). Seminal work in the psychological sciences has demonstrated the association between affective bias and depressive symptoms such that those with depression interpret emotional stimuli more negatively compared to non-depressed controls (Gur et al., 1992; Mathews and MacLeod, 2005; Surguladze et al., 2005; Beck, 2008; Harmer et al., 2009b; Gotlib and Joormann, 2010; Disner et al., 2011; Bijanki et al., 2014; Rutter et al., 2020). Previous studies have shown that in depressed patients taking antidepressant medication, behavioral ratings of emotionally valence facial expression stimuli can predict both, future changes in mood and antidepressant response to the medication given (Harmer et al., 2009b,a, 2004; Roiser et al., 2012; Harmer, 2013).

## 2 Materials and methods

### 2.1 Study overview

#### 2.1.1 Emory cohort

**Figure 1:**
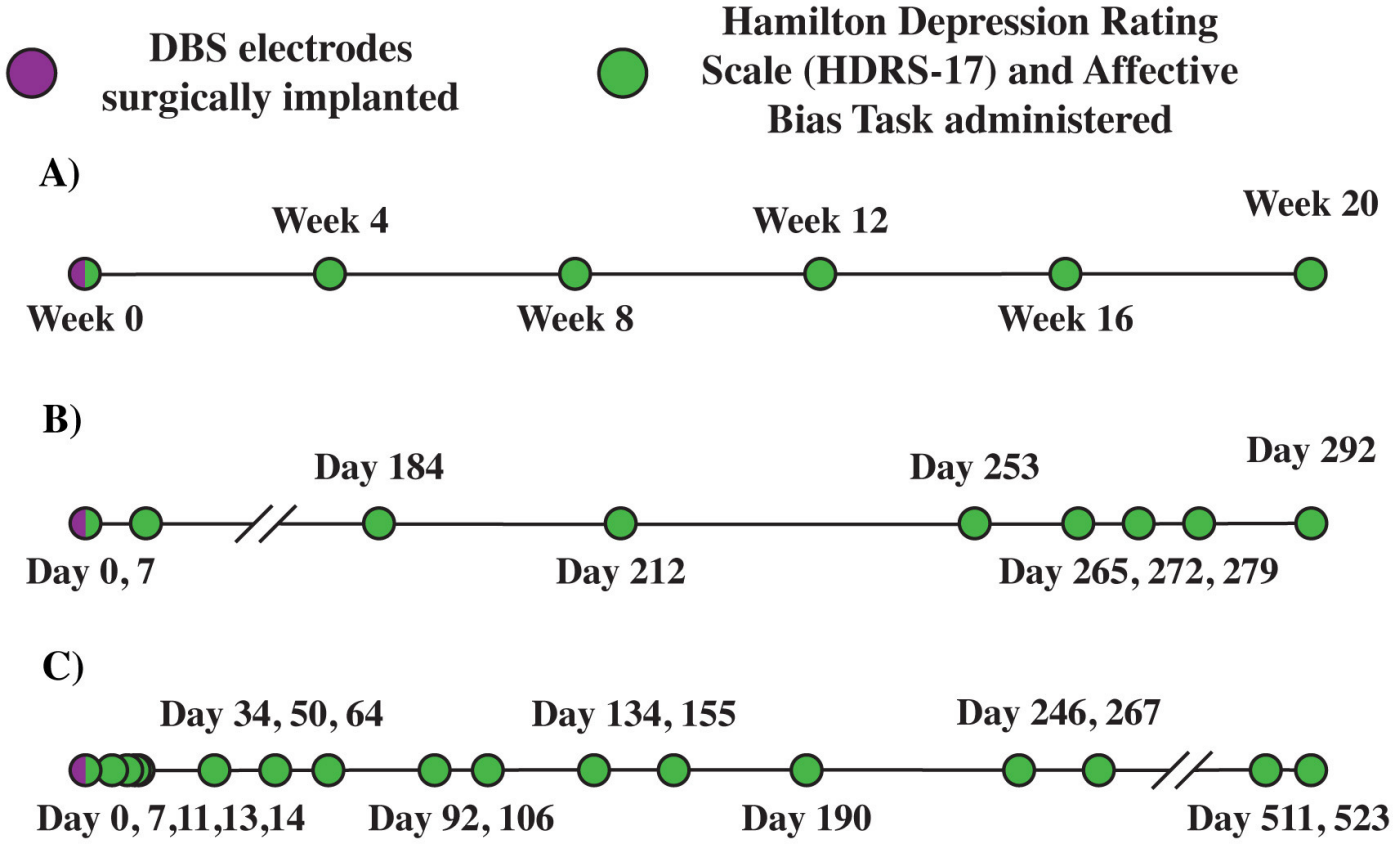
Deep brain stimulation (DBS) timeline. **(A)** Emory E001. **(B)** Baylor B001 (COVID-19 disruptions). **(C)** Baylor B002 (patient paused research activities). Example Emory **(A)** and Baylor **(B, C)** patient deep brain stimulation timelines. B001 timeline **(B)** Depicts impact of COVID-19 disruptions on the study. B002 timeline **(C)** Depicts impact of study disruptions resulting from patient pausing research activities.

This study was approved by the Emory University Institutional Review Board (clinicaltrials.gov NCT00367003) and the FDA (IDE: G060028). Data was collected from 2015 to 2018. In this clinical trial, eight participants with TRD underwent surgical implantation of DBS electrodes, followed by open-label programming of deep brain stimulation (DBS) over 6 months, with the primary clinical outcome measure being the change in depression symptom severity from the beginning of this period, as measured through the Hamilton Depression Rating Scale (HDRS-17) and other clinical assessments. These participants are referred to as E001-E008. In addition to their clinical depression rating scales, participants also completed the Affective Bias Task (ABT) at each out-patient visit to the psychiatry clinic, approximately once per month (Figure 1A).

#### 2.1.2 Baylor cohort

This study was approved by the Baylor College of Medicine Institutional Review Board (clinicaltrials.gov NCT03437928) and the FDA (IDE: G180300). Data were collected from 2020 to 2022. In this clinical trial, participants with TRD underwent DBS implantation and intracranial monitoring, followed by open label programming of DBS over 8 months, with the primary clinical outcome measure being the change in depression severity as measured by the HDRS-17 and MADRS. These participants are referred to as B001 and B002. B001 received DBS over 10 months due to a prolonged pause in the study (177 days) required by COVID-19 restrictions during 2020. B002 received DBS over 18 months due to a prolonged pause in the study (244 days) resulting from a temporary disruption in research activities (Figures 1B, C; Sheth et al., 2022).

### 2.2 Participants

#### 2.2.1 Emory cohort

Key inclusion criteria consisted of: age between 18 and 70 years with a diagnosis of major depressive disorder (confirmed with the Structured Clinical Interview for *DSM-IV*) and by the study psychiatrists (PRP, ALC); a current depressive episode of at least 12 months without significant response to at least four adequate antidepressant treatments (scoring three or higher on the Antidepressant Treatment History Form and verified through medical records); lifetime failure or intolerance of electroconvulsive therapy or inability to receive electroconvulsive therapy; average score ≥20 on the HDRS-17 averaged over the 4 weeks prior to surgical implant; Global Assessment of Function (GAF) of 50 or less; capacity to provide informed consent; and being able to relocate to the Atlanta area for 7 months (American Psychiatric Association, 2013). Participants joining the study were notified of the study's purpose, participant commitment, potential risks and benefits, and all patients provided written informed consent. Additional demographic and clinical characteristics of the patients can be found in Table 1.

**Table 1:** Emory cohort (*n* = 7) demographic and clinical characteristics of study participants receiving subcallosal cingulate deep brain stimulation for treatment-resistant depression.

**Subject ID**	**Gender**	**Age**	**Race**	**Psychiatric comorbidities**	**Current depressive episode chronicity (months)**	**Baseline HDRS-17**	**MDD age onset**
E001	Female	70	Non-hispanic white	None	36	21	36
E002	Male	64	Non-hispanic white			22	
E003	Female	62	Non-hispanic white			25	
E004	Male	57	Non-hispanic white	None	24	17	27
E005	Male	38	Non-hispanic white	GAD	60	18	32
E006	Female	45	Non-hispanic white	None	13	27	16
E007	Male	28	Non-hispanic white			22	

#### 2.2.2 Baylor cohort

Participants at the BCM site included one male and one female with a mean age of 46.5. Key inclusion criteria consisted of: age of 22–70 years with a *DSM-V* diagnosis of a current major depressive episode (MDE) ≥ 24 months duration without significant response to at least four adequate antidepressant treatments from at least two different treatment categories (scoring three or higher on the Antidepressant Treatment History Form and verified through medical records); previous trial of electroconvulsive therapy; average score ≥20 on the HDRS-17 on two separate assessments (at initial screening and 1 week before surgery); normal brain MRI within 3 months of surgery; stable antidepressant medication regimen for the month preceding surgery; capacity to provide informed consent; and ability and willingness to attend regular clinic visits for at least 12 months (American Psychiatric Association, 2013). Participants joining the study were notified of the study's purpose, participant commitment, potential risks and benefits, and all patients provided written informed consent. Additional demographic and clinical characteristics of the two patients can be found in Table 2.

**Table 2:** Baylor cohort (*n* = 2) demographic and clinical characteristics of study participants receiving subcallosal cingulate and ventral capsule/ventral striatum deep brain stimulation for treatment-resistant depression.

**Subject ID**	**Gender**	**Age**	**Race**	**Psychiatric comorbidities**	**Current depressive episode chronicity (years)**	**Baseline HDRS-17**	**MDD age onset**
B001	Male	37	Hispanic white	OCD	10	24	27
B002	Female	56	Non-hispanic white	None	30	22	26

### 2.3 Deep brain stimulation

#### 2.3.1 Emory cohort

**Figure 2:**
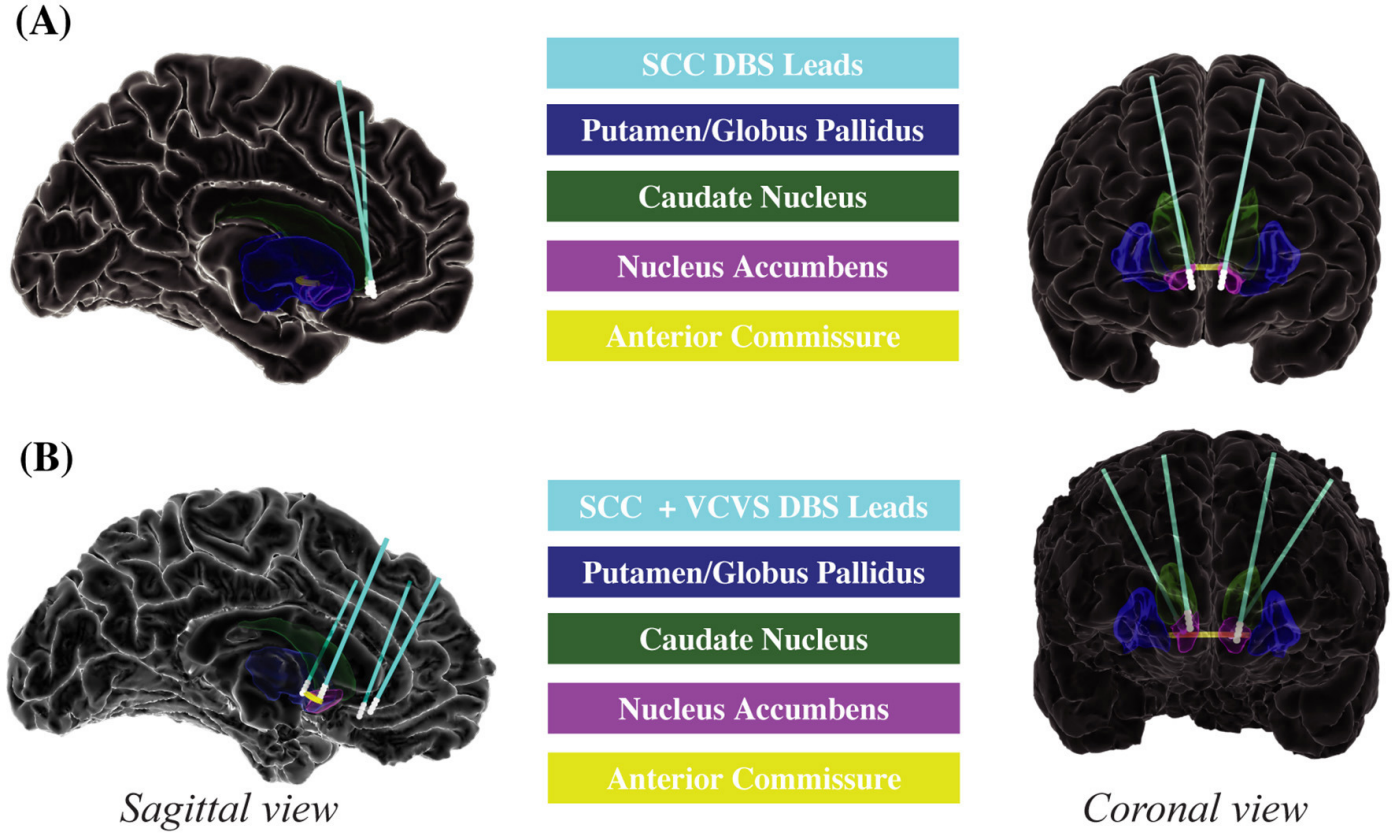
Deep brain stimulation electrode placement. **(A)** Emory. **(B)** Baylor. Sagittal and coronal views of DBS electrodes and regions of interest for Emory and Baylor patients. Emory patients **(A)** received DBS to the SCC. Baylor patients **(B)** received DBS to the SCC and VCVS. DBS, deep brain stimulation; SCC, subcallosal cingulate; VCVS, ventral capsule/ventral striatum.

DBS electrodes were surgically implanted in the subcallosal cingulate (SCC; Figure 2A). During the outpatient therapeutic phase of the study, patients received continuous stimulation to the SCC in the electrode contact combinations shown through diffusion weighted imaging (DWI) tractography and intraoperative behavioral testing to be most likely to generate a therapeutic response (Riva-Posse et al., 2018; Sendi et al., 2021). Participants' stimulation was consistently administered at 130 Hz using the Lilly pulse waveform with passive recharge as generated by the Medtronic PC+S implantable pulse generators. Amplitude ranged from 4 to 8 mA per lead, and pulse width ranged from 87 to 91 microseconds as indicated clinically.

#### 2.3.2 Baylor cohort

DBS electrodes were surgically implanted in the subcallosal cingulate (SCC) and the ventral capsule/ventral striatum (VCVS; Figure 2B). During the outpatient therapeutic phase of the study, patients received multi-lead combinations of stimulation to the SCC and VCVS in contact combinations guided by DWI tractography and intracranial electrophysiology to be most likely to generate a therapeutic response (Sheth et al., 2022). Participants' stimulation was consistently administered at 130 Hz using the Lilly-pulse waveform with passive recharge as generated by the Boston Scientific Gevia Implantable Pulse Generator. Amplitude ranged from 4 to 8 mA per lead, and pulse width ranged from 120 to 180 microseconds as determined clinically.

### 2.4 Affective bias task

#### 2.4.1 Emory cohort

**Figure 3:**
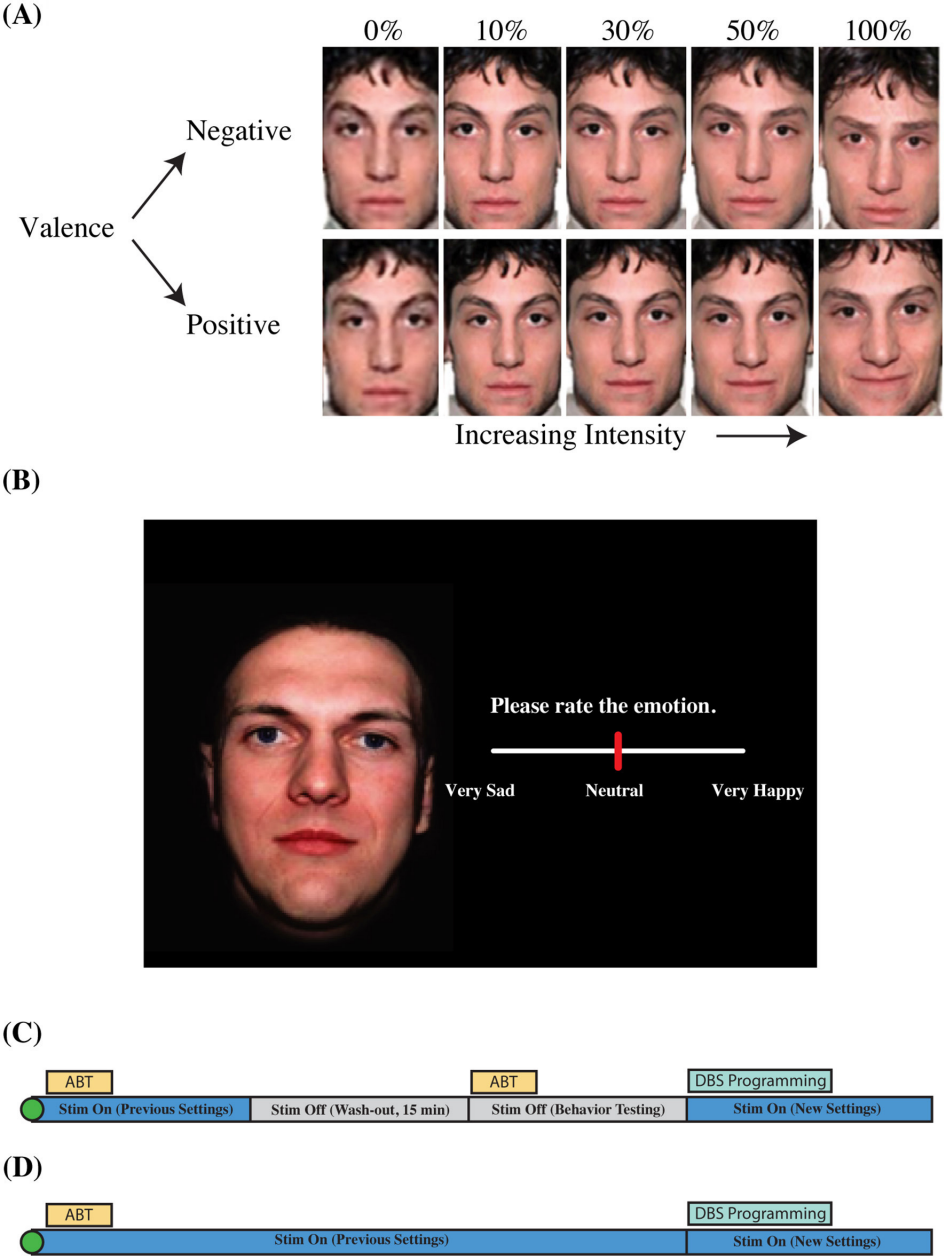
Aective bias task information and visit structure. **(A)** Morphed faces. **(B)** Task slide. **(C)** Outpatient visit structure (Emory). **(D)** Outpatient visit structure (Baylor). Visualization of valence and intensity parameters for faces shown in the aective bias task **(A)**. Faces were morphed from neutral to sad, and neutral to happy in steps of 10, 30, 50, and 100%. Faces were presented on a black background **(B)**. Participants used a slider bar to rate the emotion of the face. Outpatient visit structure shows order of behavioral inventory tasks, and changes to stimulation parameters over the 1 h visit **(C,D)**. Original images were taken from the NimStim facial expression stimulus set (Tottenham et al., 2009).

After the administration of the HDRS-17 at the beginning of each out-patient visit, participants completed the affective bias task. Participants rated the emotional content of static, colorized photographs of adult human faces presented to a display monitor (Viewsonic VP150, 1,920 × 1,080) positioned at a distance of 57 cm. Faces consisted of emotional and neutral faces adapted from the NimStim Face Stimulus Set (Tottenham et al., 2009). Happy, sad, and neutral face exemplars (six identities each; three male, three female) were morphed using a Delaunay tessellation matrix to generate subtle facial expressions ranging in emotional intensity from neutral to maximally expressive in steps of 10, 30, 50, and 100% for happy and sad faces alike (Figure 3A). The final stimulus set consisted of 54 stimulus exemplars [six identities × nine levels of intensity (100% sad, 50% sad, 30% sad, 10% sad, neutral, 10% happy, 30% happy, 50% happy, and 100% happy); Bijanki et al., 2014].

Trials began with the presentation of a white fixation cross presented on a black background for 1,000 ms (jittered +/– 100 ms) followed by the simultaneous appearance of a face and a rating prompt (appearing on the left and right sides of the display, respectively; Figure 3B). The rating prompt consisted of an active analog slider placed below text instructing patients to “Please rate the emotion” (Figure 3B). Participants used a computer mouse to indicate their rating by clicking a location on the slider bar. Ratings were recorded using a continuous scale ranging from 0 (“Very Sad”) to 0.5 (“Neutral”) to 1 (“Very Happy”). Stimuli were presented in a blocked design in which all happy faces (including neutral) appeared in one block while all sad faces (plus neutral) appeared in a separate block, from here on referred to as the positive block and the negative block, respectively. Blocks consisted of one presentation of each image for a total of 30 randomized trials per block which vary in intensity across the block (six identities × five levels of intensity). A recording session consisted of two blocks each of happy and sad faces, alternating between positive and negative blocks. The order of the blocks was not counterbalanced across participants; the negative block was presented first for each administration of the ABT. The ABT was administered approximately once each month during the 6 months of open-label programming. ABT blocks were collected twice per participant per visit (two blocks each of happy and sad faces), with one administration before transient discontinuation of stimulation, and another following acute discontinuation of stimulation (Figure 3C). Data collected from the ABT with and without stimulation were combined into a single dataset after a linear mixed-effects model confirmed the non-significant effects of transient discontinuation of stimulation on ratings of faces (for further information, see Results: Stimulation Status Effect on Bias Ratings). All stimuli were presented using the Psychtoolbox extensions for MATLAB (Brainard, 1997).

#### 2.4.2 Baylor cohort

The HDRS-17 and ABT were administered to the TRD patients using the same protocol as the Emory cohort except for the following differences. The ABT was administered three times per participant per visit (three blocks each of happy and sad faces), with all administrations of the ABT performed under active stimulation except for one visit at which the ABT is administered without stimulation prior to surgery (Figure 3D). In addition, the order of the positive and negative blocks of the ABT was counterbalanced across participants. We intended to administer the ABT once every 2 weeks for the first 5 months of open-label programming. However, due to COVID-19 complications, we were not able to collect data at our intended frequency. Data were collected at variable intervals for both B001 (7–177 days between visits) and B002 (14–28 days between visits; Figure 1). Data was collected at variable intervals for B001 and B002 (Figures 1B, C for a detailed timeline).

### 2.5 Normative data collection for affective bias task

To obtain a normalized measure of ABT performance when performed by healthy controls, we used Amazon's Mechanical Turk (MTurk) platform to administer the ABT to healthy, non-depressed individuals. The stimuli and experimental design were identical to the task administered to TRD patients with a few exceptions. Given the nature of the task, we were unable to control the distance from or the size of the screen. We were also unable to add jitter between trials. Images were the same as the TRD studies and were presented in pseudo-random order in blocked design (i.e., neutral plus sad faces in one block, neutral plus happy faces in a separated block) in the same way as in the TRD studies. Four hundred MTurk participants were initially screened for depression symptoms, and the face rating task was administered to the participants exhibiting no initial symptoms via Qualtrics (*n* = 86, Mean Age = 39.3, M:F ratio = 47:39). Averaged “norm” values were calculated for each face after administration of the task to the healthy controls through MTurk (the “expected score”). To control for outliers and address the possibility of poor task performance, we excluded the small number of ratings that were more than two standard deviations outside the mean for each image. Those values were then subtracted from the TRD patients' ratings of the same faces (the “observed score”) to determine each patient's deviance from the norm value for each face. This measure is referred to as the bias score, which we use as an operationalized measure of negative affective bias.

### 2.6 Statistical approach

#### 2.6.1 Pre-DBS to post-DBS affective bias scores

The affective bias score change between pre-DBS and post-DBS timepoints was identified at the group level (i.e., all trials from all patients) using MATLAB 2023b. Pre-DBS scores were defined as those taken at the first out-patient visit before stimulation was turned on. Two subjects did not have an ABT score from that appointment, so the score collected closest in time to the first outpatient visit but before stimulation was turned on was used. Post-DBS scores were defined as scores taken on the final outpatient visit. Student's two-sample *t*-tests (*t*-test 2, MathWorks 2023b) were used to identify if there were differences between pre- and post-DBS ABT scores for total bias (positive and negative valences across all intensities except neutral), positive bias (all positive trials at all intensities except neutral), and negative bias (all negative trials at all intensities except neutral). Permutation testing was used to assess the robustness of the group level comparisons, where ABT scores were shuffled between the pre- and post- conditions, and the new pseudo pre- and post-DBS distributions were compared using a student's two-sample *t*-test for 5,000 iterations, and a permutation *p*-value was computed by dividing the number of pseudo comparison t-statistics that were more extreme than the true t-statistic by the total number of iterations [permutation *p*-value = (# pseudo t-statistics <= true t-statistic)/5,000].

#### 2.6.2 Linear modeling

Data analysis was performed using RStudio (RStudio Team, 2020). Linear modeling was done at the group level (i.e., data from all 10 patients across both clinical sites were entered into the same model). The final dataset, which included bias scores matched with same-day longitudinal HDRS-17 scores for all 10 participants, was entered into two iterations of an “HDRS~Bias” linear mixed-effects model (Bates et al., 2015). For the first iteration of the linear mixed-effects model, the dependent variable was the HDRS-17 score, with fixed effects of “weeks since surgical implantation of DBS electrodes,” bias score, valence (happy and sad), intensity (subtle and overt), and “initial HDRS-17 score just after surgical implantation of DBS electrodes,” and a random intercept by participant. Previous studies have reported differential processing between emotionally positive and negative stimuli. Thus, we fitted an interaction between the bias score and valence fixed effects (Levens and Gotlib, 2010; Rottenberg et al., 2005). Of note, the data set for participant E005 was missing the initial HDRS-17 score and was excluded from this first iteration of the model. The “initial HDRS-17 score just after surgical implantation of DBS electrodes” yielded a non-significant result (*p* = 0.52), so a second linear mixed-effects model was run which included data from all 10 participants and, aside from the exclusion of “initial HDRS-17 score just after surgical implantation of DBS electrodes,” included the same fixed effects, interactions, and random effects. Each iteration of the model initially included a random intercept effect by cohort, but the random effect was removed due to a singular fit within the model. A Type II Wald chi-square ANOVA test was then performed on the final iteration of the linear mixed-effects model (Table 3). The same linear modeling analyses were applied to each cohort (Baylor, Emory) separately (Tables 4, 5).

**Table 3:** Type II Wald chi-square ANOVA test results for combined cohort linear mixed-effects model.

**Fixed effect**	**c^2^**	**Df**	**Pr (>c^2^)**
Bias score	10.49	1	0.0012
Valence	0.03	1	0.8711
Bias score^*^ Valence	8.47	1	0.0036
Week	489.65	1	<0.0001
Intensity	0.02	1	0.9022

Week, weeks since surgical implantation of deep brain stimulation electrodes. The ^*^ denotes an interaction of fixed effects.

**Table 4:** Type II Wald chi-square ANOVA test results for Baylor-only linear mixed-effects model.

**Fixed effect**	**c^2^**	**Df**	**Pr (>c^2^)**
Bias score	32.64	1	<0.0001
Valence	1.38	1	0.2405
Bias score^*^ Valence	0.0005	1	0.9830
Week	64.1889	1	<0.0001
Intensity	0.5228	1	0.4697

Week, weeks since surgical implantation of deep brain stimulation electrodes. The ^*^ denotes an interaction of fixed effects.

**Table 5:** Type II Wald chi-square ANOVA test results for Emory-only linear mixed-effects model.

**Fixed effect**	**c^2^**	**Df**	**Pr (>c^2^)**
Bias score	6.06	1	0.0138
Valence	0.0195	1	0.8889
Bias score^*^ Valence	2.6278	1	0.1050
Week	1653.82	1	<0.0001
Intensity	0.1589	1	0.6902

Week, weeks since surgical implantation of deep brain stimulation electrodes. The ^*^ denotes an interaction of fixed effects.

#### 2.6.3 Leave-one-out cross-validation (LOOCV)

LOOCV was performed at the patient level using the same model structure as is reported in the linear mixed-effects analyses. We held out one patient at a time, refit the LME, and used this model to predict HDRS-17 scores per subject. We then computed the *R*^2^ between the predicted and actual HDRS scores at each week per subject, and performed a single-sample student's *t*-test to compare the *R*^2^ values across patients to zero.

#### 2.6.4 Control analysis: stimulation status effect on bias scores

For the Emory cohort, ABT blocks (*n* = 146) were collected both with (*n* = 73) and without (*n* = 73) active deep brain stimulation. For the Baylor cohort, stimulation was chronically active except for one administration of the ABT from the first visit. A separate linear mixed-effects model was performed to determine if stimulation status could be excluded from the fixed effects of the initial HDRS~Bias linear model described in the previous section. Due to the limited sample of Baylor cohort ABT administrations performed without stimulation, a control analysis was performed using data from the Emory cohort to determine if stimulation status had a significant effect on bias ratings from the ABT. A Bias~Stim linear mixed-effects model predicting bias scores was fitted with fixed effects of stimulation status, “days since surgical implant of DBS electrodes,” valence, and intensity, with a random intercept for participant.

## 3 Results

**Figure 4:**
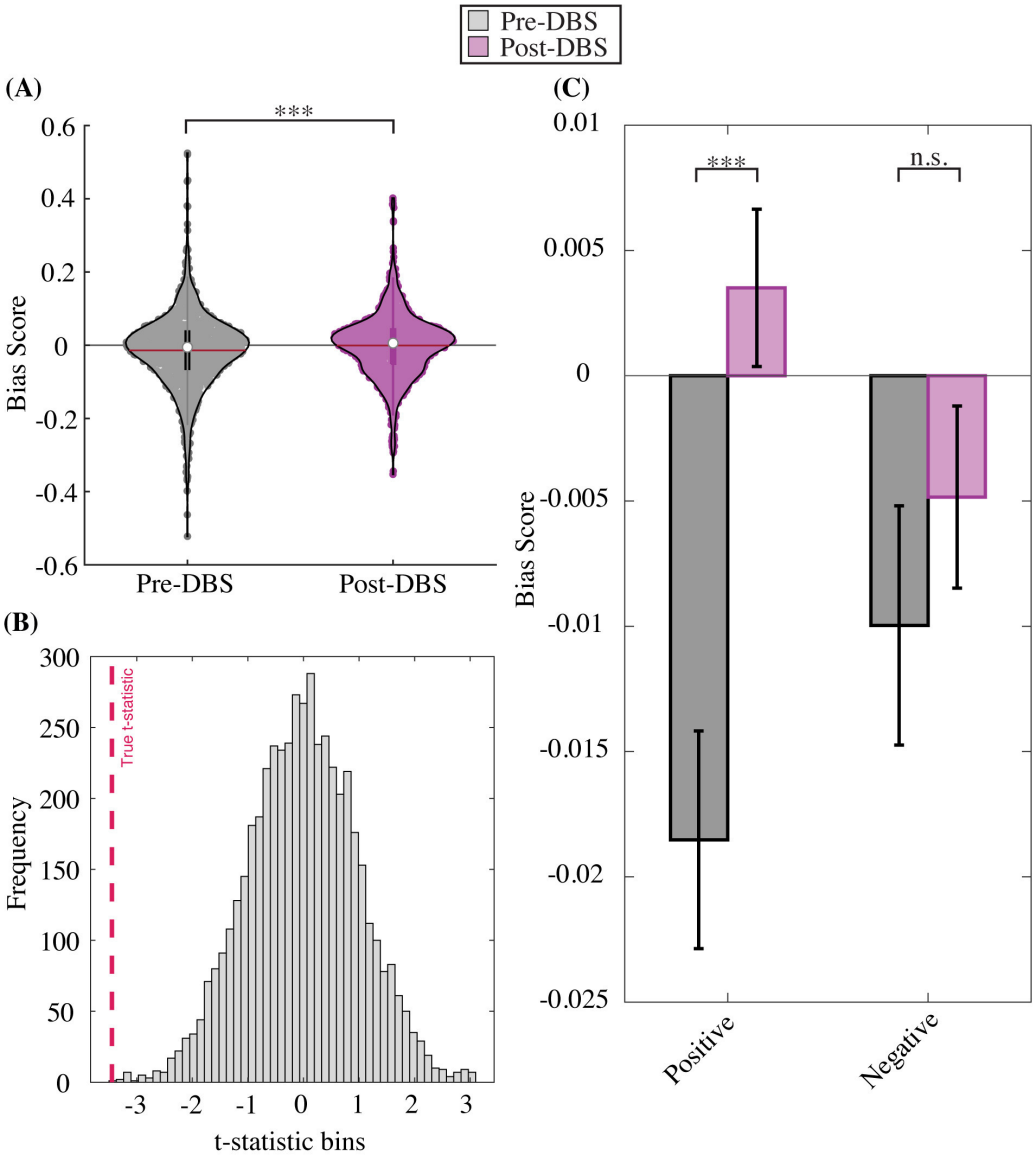
Pre- to post- DBS change in affective bias score. **(A)** Trial-level total bias change. **(B)** Permutation test distribution. **(C)** Trial-level bias change by valence. Total bias (across positive and negative trials and all intensities) becomes less negative on average after DBS compared to before DBS onset **(A)**. Permutation tests demonstrate the robustness of total bias shift between pre- to post-DBS timepoints, where the true distribution difference is more extreme than shuffled pre- and post-DBS distribution differences **(B)**. Total bias distributions are separated by valence (positive, negative), which shows a significant pre- to post-DBS bias change for the positive but not negative stimulus condition **(C)**.

We first sought to examine if ABT scores changed between the longitudinal pre- and post-DBS timepoints (see methods for pre- and post- timepoint definitions). To do so, we examined average ABT scores across all trials and all subjects for total, positive, and negative bias conditions. The total average ABT score across both valences (positive, negative) and all intensities showed a positive change between pre- and post-DBS timepoints, such that the average negative affective bias seen before the onset of chronic DBS was almost entirely extinguished, with a final total bias score approaching zero [t_(2, 338)_ = −3.4456, *p* = 5.80e-04; Figure 4A]. This result was further qualified by a permutation test showing the t-statistic of our true distributions was statistically significant compared to randomly shuffled pre- and post- distributions (*p* < 0.0001; Figure 4B). When positive and negative trials were separated, we found that the negative valence condition showed no change between pre- and post-DBS timepoints [t_(1, 168)_ = −0.8699, *p* = 0.3845; Figure 4C]. Positive valence trials on the other hand, showed a significant positive change in ABT scores, such that an initial negative bias seen before chronic DBS was ameliorated, and even became positively biased, after DBS [t_(1, 168)_ = −4.2128, *p* = 2.716e-05; Figure 4C], which was recapitulated by permutation testing (*p* < 0.0001, Supplementary Table S1).

Our next objective across both cohorts was to determine whether affective bias scores vary as a function of HDRS-17 scores at the group level. We assessed the potential relationship between HDRS-17 scores and bias scores using a group-level HDRS~Bias mixed-effects linear model with the following covariates: weeks since surgical implantation of DBS electrodes, bias score, valence (happy and sad), intensity (subtle and overt), an interaction term between bias score and valence, and a random intercept by participant (Supplementary Table S2). The model reveals a significant interaction of bias rating by valence, indicating a statistically robust relationship between bias scores and HDRS-17 scores, while statistically controlling for the effects of time, patient variation, and initial depression severity. Type II Wald chi square ANOVA revealed a significant fixed-effect for the bias score [fixed-effect estimate = −0.26, Chi square = 10.49, Pr (>χ^2^) = 0.0012; Table 3] such that more negative bias scores were associated with higher HDRS-17 scores. The Type II Wald chi-square ANOVA test also resulted in a significant interaction between bias scores and valence [fixed-effect estimate = −2.88, Chi square = 8.47, Pr (>χ^2^) = 0.0036; Table 3] such that bias scores collected from positive blocks of the ABT were stronger predictors of HDRS-17 scores than bias scores collected from negative blocks of the ABT. Lastly, the Type II Wald chi square ANOVA test resulted in a significant fixed-effect for weeks since surgical implantation of DBS electrodes [fixed-effect estimate = −0.06, Chi square = 489.65, Pr (>χ^2^) < 0.0001; Table 3] such that greater time elapsed in the study was associated with a decrease in HDRS-17 scores.

A parallel analysis was applied to each cohort (Emory, Baylor), separately to examine the robustness of the results across clinical sites. The type II Wald chi square ANOVA for the Baylor cohort revealed a significant effect for bias score [fixed-effect estimate = −3.05, Chi square = 32.64, Pr (>χ^2^) < 0.0001; Table 4], such that more negative bias scores were associated with higher HDRS-17 scores, and with weeks since surgical implantation [fixed-effect estimate = −1.47, Chi square = 64.19, Pr (>χ^2^) < 0.0001; Table 4], such that greater time elapsed in the study was associated with a decrease in HDRS-17 scores. This finding was echoed in the Emory cohort for both bias score [fixed-effect estimate = −0.70, Chi square = 6.06, Pr (>χ^2^) < 0.014; Table 5] and weeks since implantation [fixed-effect estimate = −0.32, Chi square = 1653.82, Pr (>χ^2^) < 0.0001; Table 5].

To further validate our findings, we conducted a leave-one-(patient)-out cross-validation (LOOCV) analysis to predict HDRS-17 scores from bias scores while incorporating the effects of valence, intensity, and week as in the aforementioned linear model for the combined cohort dataset (see methods). Results suggest that the mixed effects of bias score, valence and intensity explain a moderate amount of the variability in HDRS scores per week within each subject [M_R − squared_ (SD) = 0.46(0.28); T_(9)_ = 5.04, *p* < 0.001].

### 3.1 Control analysis: stimulation status effect on bias ratings

To determine if stimulation status could be excluded from the fixed effects of the HDRS~Bias linear model, a separate linear mixed-effects model was fitted to determine if stimulation status had a significant effect on bias scores (Bias~Stim). This resulted in the following linear mixed-effects model: [Bias Score ~ Stimulation Status + “Days Since Surgical Implant of DBS Electrodes” + Valence + Intensity + (1|Subject ID); Supplementary Table S1]. There was not a significant effect of stimulation status on bias scores (*p* = 0.53), so the fixed effect of stimulation status was excluded from our linear mixed-effects model predicting HDRS-17 scores (for further information, see Methods: Linear Modeling).

## 4 Discussion

Previous studies have demonstrated modulation of affective bias in depressed patients undergoing oral antidepressant treatment (Harmer et al., 2004, 2009a,b; Roiser et al., 2012; Harmer, 2013). Here we report the first instance in which this effect is observed in patients with treatment-resistant depression, and those undergoing deep brain stimulation. Specifically, we show that affective bias scores track with clinical depression evaluations over the longitudinal time course of DBS therapy in patients studied at two academic medical centers. Furthermore, we demonstrate the applicability of affective bias as a read-out of long-term DBS therapy.

The efficacy of DBS has been difficult to establish in clinical trials: reports show highly-variable patient response (20–60%) and remission rates (9–50%) over a wide range of time periods (3 months – several years) in chronic stimulation targeting the SCC or VC/VS, with some studies showing comparable “response rates” between active and sham stimulation groups (Lozano et al., 2008; Delaloye and Holtzheimer, 2014). We believe a substantial portion of this variability could be ameliorated by shifting the focus away from self-reported mood inventories which rely on a patient's insight into their own emotional state, which is often impaired in mood disorders such as depression and can be easily influenced by external factors relating to the study. For example, depressed patients are more likely to suffer from alexithymia, a condition associated with difficulty describing one's own emotional experiences (Taylor, 1984; Hemming et al., 2019). Even healthy non-depressed patients are known to have difficulty remembering their affective experiences accurately over time, an effect which is exaggerated MDD populations (Köhler et al., 2015; Talarowska et al., 2016). Furthermore, in clinical DBS trials for TRD, factors such as the greater cost or invasiveness of DBS can lead to changes in self-reports of mood that result from placebo effects and do not necessarily reflect the patients' true emotional states (Davidson et al., 2020). Demand characteristics inherent to a voluntary clinical DBS trial for treatment-resistant depression can also result in changes in a patient's self-report of mood to fit the goals of the study, and a patient's own expectations and optimism about the DBS treatment can result in subconscious changes in self-reports of mood that do not reflect true changes in their emotional state (Davidson et al., 2020). These inherent limitations have given rise to a search for biomarkers of treatment response, including electrophysiological signatures (Alagapan et al., 2023), peripheral nervous system markers (Riva-Posse et al., 2018), and as in the current report, cognitive proxies (Hilimire et al., 2015).

Given the limitations of the patient-reported depression scales, there exists a need for a measurement of mood that can exceed the reliability of symptom reports of mood and move closer to the cognitive or perceptual substrates underlying depressed mood. As a subconscious account of mood biases that can be administered in minutes, the ABT meets the first criterion for an effective cognitive marker of mood. Further, the findings from this study provide proof-of-concept for using the ABT behavioral assessment as a proxy of longitudinal changes in depression for evaluation of therapeutic effects. These findings support the future goal of using the affective bias task as a means of quantifying changes not only in fine grained fluctuations in instantaneous mood, but also in the larger construct of depressive disease state.

Using the ABT in two separate cohorts of TRD patients undergoing DBS, we show a significant relationship between the progression of HDRS-17 scores throughout the DBS treatment and affective bias, such that rating emotional facial expressions as sadder than healthy controls' ratings (negative affective bias) is associated with more severe depressive symptoms as measured on the HDRS-17. This relationship was detected longitudinally with the antidepressant effects of DBS therapy and stands robustly in two separate cohorts of patients across two clinical trials taking place at separate clinical sites, regardless of stimulation target location (SCC-only or SCC + VCVS) and clinical team. Even further, cross-validation modeling suggested that the combined effects of bias score, valence, and intensity explained a moderate and significant proportion of the variability in HDRS scores at each week within each subject.

To further characterize the nature of this relationship, we also assessed if this relationship was modulated by other factors such as the valence (happy vs. sad) and intensity (subtle vs. overt) of the faces from which bias scores were collected. Although we observed no consistent pattern that suggests the relationship between bias scores and HDRS-17 scores is modulated by intensity, we observed a significant interaction between bias scores and valence such that bias scores collected from positive blocks of the ABT were more strongly correlated with depressive severity. This finding suggests that it may be more effective to track long-term mood changes in depressed populations undergoing DBS by using positive stimuli rather than negative stimuli, which is in line with previous studies suggesting that normative positive rather than negative affective processing is essential for optimal emotional processing (Fredrickson, 2004; Schwartz and Caramoni, 1989), and that focusing on positive emotional stimuli during neuromodulation can attenuate negative affective biases (Young et al., 2017). Importantly, the difference in effect seen between positive and negative affective processing in this study may also clue to the distinct circuits underlying affective dysregulation in depression, and subsequently, which circuits are most affected during stimulation-based therapeutics. While positive and negative affective circuits largely overlap, the top-down activation in prefrontal cortices is most strongly associated with the generation of positive affect toward desired goals through the approach system, and particularly in the left hemisphere, whereas the bottom-up activation of the amygdala is proposed to organize negative affective processing in response to threatening or aversive cues (Davidson, 2001; Ochsner et al., 2009). Another recent study by our group found that deep-brain stimulation to the SCC restored normal amygdalar response profiles to positive facial stimuli but not to negative facial stimuli, and postulated that DBS works to release the top-down inhibition of this circuit by the prefrontal cortex to improve response to positive stimuli mediated by the top-down circuit (Fan et al., 2024). While the previous study by Fan et al. (2024) did not report behavioral effects of DBS on affective bias scores, the physiological findings from this study, and the proposed underlying mechanism of affective biases in treatment-resistant depression, align with the behavioral effects seen in the current longitudinal study. Overall, the results presented here may suggest that DBS specifically acts upon the top-down affective circuit which is most heavily implicated in positive affective processing, but not the bottom-up affective circuit which is more often associated with negative affective processing. Future studies should also examine potential differences in affective valence biases on a more acute time scale and in a larger cohort of subjects.

### 4.1 Limitations and future directions

The findings of this study suggest that our ABT is a robust cognitive proxy of mood that can be tracked longitudinally. However, this study is limited in both temporal resolution of data collection and patient sample size. Identifying an index for mood that is independent of self- and clinician- reported assessments is an essential need of a successful treatment biomarker—one that this study suggests is filled by the ABT. The second essential component of a successful treatment biomarker is increased temporal specificity for tracking the effects of treatments like DBS. While the ABT has the potential to fill this need, the task data was collected at the same interval as the clinician-administered HDRS-17 scores. Thus, while we can resolve that ABT tracks mood as effectively as the HDRS-17, we cannot examine if changes in ABT due to different stimulation parameters occur on a faster time scale than the robust changes in HDRS-17 score. Future studies should investigate if ABT can track mood similarly to other depression inventories that can be collected at shorter time intervals such as the Computerized Adaptive Test–Depression Inventory (CAT-DI; Gibbons et al., 2012), or if ABT scores change on a faster time scale than HDRS-17. One way to identify the response latency of ABT is sampling at a greater number of time points throughout the course of treatment, perhaps by using an app-based collection of the ABT data which would be more accessible to patients for day-to-day screening than commuting to an office for behavioral monitoring.

Ideally, changes in ABT would occur on the order of minutes following changes in stimulation parameters or when effective stimulation is turned off, in which case there is no need to collect ABT scores more frequently than the HDRS-17 scores. Unfortunately, this was not reflected in the current dataset, as an analysis of the effect of stimulation status on bias scores for the Emory cohort yielded a non-significant result, indicating that ABT score did not change after turning off stimulation during the Emory outpatient visits. For this reason, stimulation status was excluded from the final iteration of the linear mixed-effect model. It is possible that this relationship between bias score and stimulation status, as well as the relationship between HDRS-17 score and bias intensity (subtle vs. overt) went undetected because the study was not adequately powered to identify these effects with a sample size of 10 DBS patients. Still, it is also possible that the brief wash-out duration allotted between stimulation parameter change and ABT data collection (15 min) within an outpatient session was insufficient for the ABT score to change and for that change to be detected. The true duration of stimulation effect wash-out is not known, though some evidence suggests a slower wash-out with SCC stimulation than for VCVS (Holtzheimer et al., 2017).

In any case, these effects warrant further psychometric investigation in larger normative populations and at an increased number of time points allowing for a longer wash-out period throughout the course of treatment before implications can be fully explored in the disease population. Future studies with higher power can further explore moderating factors of the predictive relationship between bias scores from the ABT and HDRS-17 scores, as well as the extent to which the ABT can show content validity beyond convergence with canonical scales. Additional routes of exploration include studying the effect of different stimulation parameters—such as amplitude and DBS contact locations—on bias scores and depression severity, or by examining the effects of demographic factors on the reported effect. For example, age is positively associated with a fading negative affective bias, where younger patients typically show a negative affective bias, and older adults show a bias toward positive information which is hypothesized to be driven by increased emotional regulation in later life (Marsh and Crawford, 2024). The small sample size of the current study was accompanied by a disproportionate age distribution between the DBS cohorts [M(13.63) = 50.89] and the non-clinical MTurk cohort [M(13.5) = 39.3], where the MTurk cohort was significantly younger than the DBS cohorts [t_(93)_ = 2.31, *p* = 0.023]. Given that the MTurk cohort was utilized to identify a normalized measure of affective bias for comparison to the DBS cohorts, it is possible that the reported age difference affected our results. However, given that the older DBS cohort may have been pre-disposed to a higher positive score at baseline compared to our control dataset, this may suggest that the movement toward positive affect after stimulation that is detected in the current study is likely much smaller in magnitude than it would be for a younger cohort which may start at a more negative affective baseline value. Future investigation in a larger cohort which also controls for age may, in fact, find a stronger and more robust difference in affective bias with stimulation, or may allow for the detection within the shorter washout period after stimulation for which the current study was unable to detect any change. These future studies should also account for other factors that influence patient performance on the ABT including sex, ethnicity, and socioeconomic status.

## 5 Conclusion

In this study, we report a significant relationship between HDRS-17 scores and bias scores from our ABT in two separate cohorts of patients undergoing DBS for TRD. When these patients rated emotional faces shown in our ABT more negatively than non-depressed controls performing the same task, it was associated with greater depression severity. Although a similar effect has been shown with depressed patients taking oral antidepressants, to our knowledge, we report the first instance in which this effect is observed in patients undergoing DBS for TRD. Our findings represent a proof-of-concept for the potential use of our ABT as a cognitive proxy of mood that can be used to quickly evaluate the efficacy of different stimulation parameters within the growing field of neuromodulation for the purpose of treating mood disorders.

## Data Availability

The raw data supporting the conclusions of this article will be made available by the authors, without undue reservation.

## References

[B1] Alagapan S. Choi K. S. Heisig S. Riva-Posse P. Crowell A. Tiruvadi V.. (2023). Cingulate dynamics track depression recovery with deep brain stimulation. Nature 622, 130–138. 10.1038/s41586-023-06541-3 37730990 PMC10550829

[B2] American Psychiatric Association (2013). Diagnostic and Statistical Manual of Mental Disorders, 5th ed. Arlington, VA: American Psychiatric Publishing, Inc. 10.1176/appi.books.9780890425596

[B3] Bates D. Mächler M. Bolker B. Walker S. (2015). Fitting linear mixed-effects models using lme4. J. Stat. Softw. 67, 1–48. 10.18637/jss.v067.i01

[B4] Beck A. T. (2008). The evolution of the cognitive model of depression and its neurobiological correlates. Am. J. Psychiatry 165, 969–977. 10.1176/appi.ajp.2008.08050721 18628348

[B5] Bijanki K. R. Kovach C. K. McCormick L. M. Kawasaki H. Dlouhy B. J. Feinstein J.. (2014). Case report: stimulation of the right amygdala induces transient changes in affective bias. Brain Stimulat. 7, 690–693. 10.1016/j.brs.2014.05.005 24972588 PMC4167906

[B6] Brainard D. H. (1997). The psychophysics toolbox. Spat. Vis. 10, 433–436. 10.1163/156856897X00357 9176952

[B7] Burn D. J. Tröster A. I. (2004). Neuropsychiatric complications of medical and surgical therapies for Parkinson's disease. J. Geriatr. Psychiatry Neurol. 17, 172–180. 10.1177/0891988704267466 15312281

[B8] Campbell M. C. Black K. J. Weaver P. M. Lugar H. M. Videen T. O. Tabbal S. D.. (2012). Mood response to deep brain stimulation of the subthalamic nucleus in Parkinson's disease. J. Neuropsychiatry Clin. Neurosci. 24, 28–36. 10.1176/appi.neuropsych.11030060 22450611 PMC3354989

[B9] Crowell A. L. Riva-Posse P. Holtzheimer P. E. Garlow S. J. Kelley M. E. Gross R. E.. (2019). Long-term outcomes of subcallosal cingulate deep brain stimulation for treatment-resistant depression. Am. J. Psychiatry 176, 949–956. 10.1176/appi.ajp.2019.18121427 31581800

[B10] Daneshzand M. Faezipour M. Barkana B. D. (2018). Robust desynchronization of Parkinson's disease pathological oscillations by frequency modulation of delayed feedback deep brain stimulation. PLoS ONE 13:e0207761. 10.1371/journal.pone.0207761 30458039 PMC6245797

[B11] Davidson B. Gouveia F. V. Rabin J. S. Giacobbe P. Lipsman N. Hamani C. (2020). Deep brain stimulation for treatment-resistant depression: current status and future perspectives. Expert Rev. Med. Devices 17, 371–373. 10.1080/17434440.2020.1753503 32268822

[B12] Davidson R. J. (2001). The neural circuitry of emotion and affective style: prefrontal cortex and amygdala contributions. Soc. Sci. Inf. 40, 11–37. 10.1177/053901801040001002

[B13] Delaloye S. Holtzheimer P. E. (2014). Deep brain stimulation in the treatment of depression. Dialogues Clin. Neurosci. 16, 83–91. 10.31887/DCNS.2014.16.1/sdelaloye 24733973 PMC3984894

[B14] Disner S. G. Beevers C. G. Haigh E. A. P. Beck A. T. (2011). Neural mechanisms of the cognitive model of depression. Nat. Rev. Neurosci. 12, 467–477. 10.1038/nrn3027 21731066

[B15] Dorz S. Borgherini G. Conforti D. Scarso C. Magni G. (2004). Comparison of self-rated and clinician-rated measures of depressive symptoms: a naturalistic study. Psychol. Psychother. 77, 353–361. 10.1348/1476083041839349 15355586

[B16] Drobisz D. Damborská A. (2019). Deep brain stimulation targets for treating depression. Behav. Brain Res. 359, 266–273. 10.1016/j.bbr.2018.11.004 30414974

[B17] Fan X. Mocchi M. Pascuzzi B. Xiao J. Metzger B. A. Mathura R. K.. (2024). Brain mechanisms underlying the emotion processing bias in treatment-resistant depression. Nat. Mental Health. 2, 583–592. 10.1038/s44220-024-00238-w37693557

[B18] Fenoy A. (2020). Challenges in deep brain stimulation for depression. Rev. Bras. Psiquiatr. 42, 347–348. 10.1590/1516-4446-2020-0878 32374796 PMC7430396

[B19] Fredrickson B. L. (2004). The broaden-and-build theory of positive emotions. Philos. Trans. R. Soc. Lond. B Biol. Sci. 359, 1367–1378. 10.1098/rstb.2004.1512 15347528 PMC1693418

[B20] Funkiewiez A. Ardouin C. Caputo E. Krack P. Fraix V. Klinger H.. (2004). Long term effects of bilateral subthalamic nucleus stimulation on cognitive function, mood, and behaviour in Parkinson's disease. J. Neurol. Neurosurg. Psychiatr. 75, 834–839. 10.1136/jnnp.2002.009803 15145995 PMC1739075

[B21] Gibbons R. D. Weiss D. J. Pilkonis P. A. Frank E. Moore T. Kim J. B.. (2012). Development of a computerized adaptive test for depression. Arch. Gen. Psychiatry 69, 1104–1112. 10.1001/archgenpsychiatry.2012.14 23117634 PMC3551289

[B22] Godlewska B. R. Harmer C. J. (2021). Cognitive neuropsychological theory of antidepressant action: a modern-day approach to depression and its treatment. Psychopharmacology 238, 1265–1278. 10.1007/s00213-019-05448-0 31938879 PMC8062380

[B23] Gotlib I. H. Joormann J. (2010). Cognition and depression: current status and future directions. Annu. Rev. Clin. Psychol. 6, 285–312. 10.1146/annurev.clinpsy.121208.131305 20192795 PMC2845726

[B24] Gur R. C. Erwin R. J. Gur R. E. Zwil A. S. Heimberg C. Kraemer H. C. (1992). Facial emotion discrimination: II. Behavioral findings in depression. Psychiatry Res. 42, 241–251. 10.1016/0165-1781(92)90116-K 1496056

[B25] Hamilton M. (1960). A rating scale for depression. J. Neurol. Neurosurg. Psychiatry 23, 56–62. 10.1136/jnnp.23.1.56 14399272 PMC495331

[B26] Harmer C. J. (2013). Emotional processing and antidepressant action. Curr. Top. Behav. Neurosci. 14, 209–222. 10.1007/7854_2012_210 22566081

[B27] Harmer C. J. Goodwin G. M. Cowen P. J. (2009a). Why do antidepressants take so long to work? A cognitive neuropsychological model of antidepressant drug action. Br. J. Psychiatry 195, 102–108. 10.1192/bjp.bp.108.051193 19648538

[B28] Harmer C. J. O'Sullivan U. Favaron E. Massey-Chase R. Ayres R. Reinecke A.. (2009b). Effect of acute antidepressant administration on negative affective bias in depressed patients. Am. J. Psychiatry 166, 1178–1184. 10.1176/appi.ajp.2009.09020149 19755572

[B29] Harmer C. J. Shelley N. C. Cowen P. J. Goodwin G. M. (2004). Increased positive vs. negative affective perception and memory in healthy volunteers following selective serotonin and norepinephrine reuptake inhibition. Am. J. Psychiatry 161, 1256–1263. 10.1176/appi.ajp.161.7.1256 15229059

[B30] Heller A. S. Johnstone T. Shackman A. J. Light S. N. Peterson M. J. Kolden G. G.. (2009). Reduced capacity to sustain positive emotion in major depression reflects diminished maintenance of fronto-striatal brain activation. Proc. Natl. Acad. Sci. USA 106, 22445–22450. 10.1073/pnas.0910651106 20080793 PMC2796908

[B31] Hemming L. Haddock G. Shaw J. Pratt D. (2019). Alexithymia and its associations with depression, suicidality, and aggression: an overview of the literature. Front. Psychiatry 10:203. 10.3389/fpsyt.2019.00203 31031655 PMC6470633

[B32] Hilimire M. R. Mayberg H. S. Holtzheimer P. E. Broadway J. M. Parks N. A. DeVylder J. E.. (2015). Effects of subcallosal cingulate deep brain stimulation on negative self-bias in patients with treatment-resistant depression. Brain Stimul. 8, 185–191. 10.1016/j.brs.2014.11.010 25499035 PMC4366934

[B33] Holtzheimer P. E. Husain M. M. Lisanby S. H. Taylor S. F. Whitworth L. A. McClintock S.. (2017). Subcallosal cingulate deep brain stimulation for treatment-resistant depression: a multisite, randomised, sham-controlled trial. Lancet Psychiatry 4, 839–849. 10.1016/S2215-0366(17)30371-1 28988904

[B34] Ionescu D. F. Rosenbaum J. F. Alpert J. E. (2015). Pharmacological approaches to the challenge of treatment-resistant depression. Dialogues Clin. Neurosci. 17, 111–126. 10.31887/DCNS.2015.17.2/dionescu 26246787 PMC4518696

[B35] Köhler C. A. Carvalho A. F. Alves G. S. McIntyre R. S. Hyphantis T. N. Cammarota M. (2015). Autobiographical memory disturbances in depression: a novel therapeutic target? Neural Plast. 2015:759139. 10.1155/2015/759139 26380121 PMC4561987

[B36] Levens S. M. Gotlib I. H. (2010). Updating positive and negative stimuli in working memory in depression. J. Exp. Psychol. Gen. 139, 654–664. 10.1037/a0020283 21038984 PMC2984552

[B37] Lozano A. M. Lipsman N. Bergman H. Brown P. Chabardes S. Chang J. W.. (2019). Deep brain stimulation: current challenges and future directions. Nat. Rev. Neurol. 15, 148–160. 10.1038/s41582-018-0128-2 30683913 PMC6397644

[B38] Lozano A. M. Mayberg H. S. Giacobbe P. Hamani C. Craddock R. C. Kennedy S. H. (2008). Subcallosal cingulate gyrus deep brain stimulation for treatment-resistant depression. Biol. Psychiatry 64, 461–467. 10.1016/j.biopsych.2008.05.034 18639234

[B39] Malone D. A. Dougherty D. D. Rezai A. R. Carpenter L. L. Friehs G. M. Eskandar E. N.. (2009). Deep brain stimulation of the ventral capsule/ventral striatum for treatment-resistant depression. Biol. Psychiatry 65, 267–275. 10.1016/j.biopsych.2008.08.029 18842257 PMC3486635

[B40] Marsh C. Crawford M. T. (2024). Age is positively associated with fading affect bias: a cross-sectional comparison. Psychol. Aging 39, 139–152. 10.1037/pag0000797 38271075

[B41] Mathews A. MacLeod C. (2005). Cognitive vulnerability to emotional disorders. Annu. Rev. Clin. Psychol. 1, 167–195. 10.1146/annurev.clinpsy.1.102803.143916 17716086

[B42] Mayberg H. S. Lozano A. M. Voon V. McNeely H. E. Seminowicz D. Hamani C.. (2005). Deep brain stimulation for treatment-resistant depression. Neuron 45, 651–660. 10.1016/j.neuron.2005.02.014 15748841

[B43] Nemeroff C. B. (2007). Prevalence and management of treatment-resistant depression. J. Clin. Psychiatry 68, 17–25. 17640154

[B44] Ochsner K. N. Ray R. R. Hughes B. McRae K. Cooper J. C. Weber J.. (2009). Bottom-up and top-down processes in emotion generation: common and distinct neural mechanisms. Psychol. Sci. 20, 1322–1331. 10.1111/j.1467-9280.2009.02459.x 19883494 PMC2858766

[B45] Riva-Posse P. Choi K. S. Holtzheimer P. E. Crowell A. L. Garlow S. J. Rajendra J. K.. (2018). A connectomic approach for subcallosal cingulate deep brain stimulation surgery: prospective targeting in treatment-resistant depression. Mol. Psychiatry 23, 843–849. 10.1038/mp.2017.59 28397839 PMC5636645

[B46] Roet M. Boonstra J. Sahin E. Mulders A. E. P. Leentjens A. F. G. Jahanshahi A. (2020). Deep brain stimulation for treatment-resistant depression: towards a more personalized treatment approach. J. Clin. Med. 9:2729. 10.3390/jcm9092729 32846987 PMC7565181

[B47] Roiser J. P. Elliott R. Sahakian B. J. (2012). Cognitive mechanisms of treatment in depression. Neuropsychopharmacology 37, 117–136. 10.1038/npp.2011.183 21976044 PMC3238070

[B48] Rottenberg J. Gross J. J. Gotlib I. H. (2005). Emotion context insensitivity in major depressive disorder. J. Abnorm. Psychol. 114, 627–639. 10.1037/0021-843X.114.4.627 16351385

[B49] RStudio Team (2020). RStudio: Integrated Development for R. Boston, MA: RStudio, PBC. Available online at: http://www.rstudio.com/

[B50] Rutter L. A. Passell E. Scheuer L. Germine L. (2020). Depression severity is associated with impaired facial emotion processing in a large international sample. J. Affect. Disord. 275, 175–179. 10.1016/j.jad.2020.07.006 32734904 PMC7428842

[B51] Sackeim H. A. (2001). The definition and meaning of treatment-resistant depression. J. Clin. Psychiatry 62, 10–17. 11480879

[B52] Schwartz R. M. Caramoni G. L. (1989). Cognitive balance and psychopathology: evaluation of an information processing model of positive and negative states of mind. Clin. Psychol. Rev. 9, 271–294. 10.1016/0272-7358(89)90058-5

[B53] Sendi M. S. E. Waters A. C. Tiruvadi V. Riva-Posse P. Crowell A. Isbaine F.. (2021). Intraoperative neural signals predict rapid antidepressant effects of deep brain stimulation. Transl. Psychiatry 11:551. 10.1038/s41398-021-01669-0 34728599 PMC8563808

[B54] Sheth S. A. Bijanki K. R. Metzger B. Allawala A. Pirtle V. Adkinson J. A.. (2022). Deep brain stimulation for depression informed by intracranial recordings. Biol. Psychiatry 92, 246–251. 10.1016/j.biopsych.2021.11.007 35063186 PMC9124238

[B55] Surguladze S. Brammer M. J. Keedwell P. Giampietro V. Young A. W. Travis M. J.. (2005). A differential pattern of neural response toward sad vs. happy facial expressions in major depressive disorder. Biol. Psychiatry 57, 201–209. 10.1016/j.biopsych.2004.10.028 15691520

[B56] Talarowska M. Berk M. Maes M. Gałecki P. (2016). Autobiographical memory dysfunctions in depressive disorders. Psychiatry Clin. Neurosci. 70, 100–108. 10.1111/pcn.12370 26522618

[B57] Taylor G. J. (1984). Alexithymia: concept, measurement, and implications for treatment. Am. J. Psychiatry 141, 725–732. 10.1176/ajp.141.6.725 6375397

[B58] Temel Y. Kessels A. Tan S. Topdag A. Boon P. Visser-Vandewalle V. (2006). Behavioural changes after bilateral subthalamic stimulation in advanced Parkinson disease: a systematic review. Parkinsonism Relat. Disord. 12, 265–272. 10.1016/j.parkreldis.2006.01.004 16621661

[B59] Tottenham N. Tanaka J. W. Leon A. C. McCarry T. Nurse M. Hare T. A.. (2009). The NimStim set of facial expressions: judgments from untrained research participants. Psychiatry Res. 168, 242–249. 10.1016/j.psychres.2008.05.006 19564050 PMC3474329

[B60] Touloumis C. (2021). The burden and the challenge of treatment-resistant depression. Psychiatriki 32, 11–14. 10.22365/jpsych.2021.046 34990376

[B61] Wideman T. H. Sullivan M. J. L. Inada S. McIntyre D. Kumagai M. Yahagi N.. (2013). “Beck depression inventory (BDI),” in Encyclopedia of Behavioral Medicine, eds. Gellman M. D. Turner J. R. (New York, NY: Springer New York), 178–179.

[B62] Young K. D. Misaki M. Harmer C. J. Victor T. Zotev V. Phillips R.. (2017). Real-time functional magnetic resonance imaging amygdala neurofeedback changes positive information processing in major depressive disorder. Biol. Psychiatry 82, 578–586. 10.1016/j.biopsych.2017.03.013 28476207 PMC5610066

[B63] Zhdanava M. Pilon D. Ghelerter I. Chow W. Joshi K. Lefebvre P.. (2021). The prevalence and national burden of treatment-resistant depression and major depressive disorder in the United States. J. Clin. Psychiatry 82:20m13699. 10.4088/JCP.20m13699 33989464

