# Supplementary material for: Affective bias predicts changes in depression during deep brain stimulation therapy
Source: Front Hum Neurosci. 2025 Mar 25;19:1539857. doi: 10.3389/fnhum.2025.1539857 (PMC11977254; doi:10.3389/fnhum.2025.1539857)
Supplement: Supplementary file 1 [file Table_1.docx]

Supplementary Material

# Supplementary Tables

**Table S1.** Pre-DBS to Post-DBS Bias Score Change Permutation Tests

| **Valence** | **Number of Tests** | **Permutation P-Value** |
| --- | --- | --- |
| Total Bias (Positive and Negative) | 5000 | 2.00e-04*** |
|  |  |  |
| Positive | 5000 | 2.00e-04*** |
|  |  |  |
| Negative | 5000 | 0.3854 |

Statistical Significance: . < 0.1, * < 0.05, **<0.01, ***<0.001. Permutation p-value is calculated by identifying the number of pseudo distribution differences (p-values) that are more extreme than the true distribution t-statistic, and divided by the number of tests (i.e., permutation p-value = # extreme pseudo t-statistics/number of tests).

**Table S2.** HDRS score predicted by Affective Bias Score

| **Model** | **Model Coefficients** | **Fixed Effect** | **Estimate** | **SE** | **df** | **T-Value** | **Effect Size** | **P-Value** |
| --- | --- | --- | --- | --- | --- | --- | --- | --- |
| HDRS Score | H ~ B*V + W + I + H_0_ + (1\|Sub) | (Intercept) | 17.62 | 8.21 | 7.02 | 2.15 | 3.33 | 0.07. |
|  |  | Bias Rating | -0.53 | 0.71 | 9707 | -0.76 | -0.01 | 0.45 |
|  |  | Valence (Pos – Neg) | -2.67e-02 | 0.08 | 9706 | -0.32 | -5.05e-03 | 0.75 |
|  |  | Week | -5.29e-02 | 2.67e-03 | 9709 | -19.84 | -9.99e-03 | <2e-16*** |
|  |  | Initial HDRS | -0.24 | 0.37 | 7.02 | -0.65 | -4.55e-02 | 0.54 |
|  |  | Intensity | -1.86e-05 | 1.18e-03 | 9706 | -0.02 | -3.50e-06 | 0.99 |
|  |  | Bias Rating*Valence | -2.89 | 1.07 | 9708 | -2.72 | -5.47e-01 | 0.007** |
|  |  |  |  |  |  |  |  |  |
| HDRS Score | H ~ B*V + W + I + (1\|Sub) | (Intercept) | 12.84 | 1.06 | 9.08 | 12.10 | 2.44 | 6.58e-07*** |
|  |  | Bias Rating | -0.26 | 0.65 | 10370 | -0.40 | -5.00e-02 | 0.69 |
|  |  | Valence (Pos – Neg) | -1.62e-02 | 7.99e-02 | 10370 | -0.20 | -3.08e-03 | 0.84 |
|  |  | Week | -5.74e-02 | 2.60e-03 | 10370 | -22.13 | -1.09e-02 | <2e-16*** |
|  |  | Intensity | 1.39e-04 | 1.13e-03 | 10370 | 0.12 | 2.63e-05 | 0.90 |
|  |  | Bias Rating*Valence | -2.88 | 0.99 | 10370 | -2.91 | -0.55 | 0.0036** |
|  |  |  |  |  |  |  |  |  |
| Observed Bias Rating | B_o_ ~ S + D + V*I + (1\|Sub) | (Intercept) | 0.49 | 7.08e-03 | 10.30 | 68.55 | 5.05 | 4.85e-15*** |
|  |  | Stim Status | 1.62e-03 | 2.55e-03 | 5568 | 0.64 | 0.017 | 0.53 |
|  |  | Day | -1.68e-05 | 2.25e-05 | 5574 | -0.75 | -0.00017 | 0.46 |
|  |  | Valence | -2.30e-02 | 3.71e-03 | 5567 | -6.21 | -0.24 | 5.79e-10*** |
|  |  | Intensity | -3.44e-03 | 5.04e-05 | 5567 | -68.19 | -0.04 | <2e-16*** |
|  |  | Valence*Intensity | 7.51e-03 | 7.13e-05 | 5567 | 105.36 | 0.08 | <2e-16*** |

Statistical Significance: . < 0.1, * < 0.05, **<0.01, ***<0.001. Estimates reflect the modeled difference between factors within each fixed effect: “H” = HDRS-17 score, “H_0_” = Initial HDRS-17 score recorded before implant, “B” = Affective Bias Score (Observed – Expected), “B_o_” = Observed Bias Rating, “V” = Valence (Positive vs. Negative), “W” = Week, “I” = Intensity (Subtle vs. Overt), “Sub” = subject, “D” = Day, and “S” = stimulation status (On vs Off).
